# EJE PRIZE 2017: Hypothalamic AMPK: a golden target against obesity?

**DOI:** 10.1530/EJE-16-0927

**Published:** 2017-02-22

**Authors:** Miguel López

**Affiliations:** Department of PhysiologyNeurObesity Group, CIMUS, University of Santiago de Compostela-Instituto de Investigación Sanitaria and CIBER Fisiopatología de la Obesidad y Nutrición (CIBERobn), Santiago de Compostela, Spain

## Abstract

AMP-activated protein kinase (AMPK) is a cellular gauge that is activated under conditions, such as low energy, increasing energy production and reducing energy waste. Centrally, the AMPK pathway is a canonical route regulating energy homeostasis, by integrating peripheral signals, such as hormones and metabolites, with neuronal networks. Current evidence links hypothalamic AMPK with feeding, brown adipose tissue (BAT) thermogenesis and browning of white adipose tissue (WAT), as well as muscle metabolism, hepatic function and glucose homeostasis. The relevance of these data is interesting from a therapeutic point of view as several agents with potential anti-obesity and/or antidiabetic effects, some currently in clinical use, such as nicotine, metformin and liraglutide are known to act through AMPK, either peripherally or centrally. Furthermore, the orexigenic and weight-gaining effects of the worldwide use of antipsychotic drugs (APDs), such as olanzapine, are also mediated by hypothalamic AMPK. Overall, this evidence makes hypothalamic AMPK signaling an interesting target for the drug development, with its potential for controlling both sides of the energy balance equation, namely feeding and energy expenditure through defined metabolic pathways.

## Invited Author’s profile


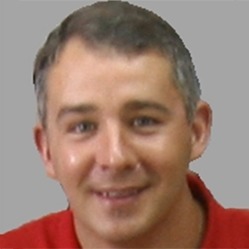


**Dr Miguel López** PhD is currently Associate Professor in Department of Physiology at the School of Medicine and the Research Centre of Molecular Medicine and Chronic Diseases (CIMUS) of the University of Santiago de Compostela, Spain. His research has focused on the regulation of energy balance and obesity, with his current interest on hypothalamic AMPK and energy sensing in the modulation of energy balance and metabolism.

## Living organisms need to sense energy

The survival of living organisms is due to their continuous exchange of energy with the environment. Intracellularly, there are thousands of different metabolic processes that underlie energy production and utilization. Heterotrophs, such as mammals, obtain energy from organic compounds, mainly carbohydrates, fats and proteins, which are oxidized to release energy that is then stored as ATP. Indeed, every living cell can be considered to contain an energy-storing ‘battery’, the main components being ATP and ADP, interconverted by the reaction ATP ↔ ADP + P. Discharging the battery therefore leads to an increase in intracellular ADP levels. As the reaction is reversible (2ADP ↔ ATP + AMP), AMP levels rise markedly when the ADP:ATP ratio increases during the energy consumption. Thus, under conditions of insufficient intracellular energy, there is an associated rise in AMP levels. An efficient evolutionary criterion for a functional intracellular energy gauge would therefore be necessary to sense the ratio of either ADP:ATP or AMP:ATP (1, 2, 3, 4).

## AMPK and the regulation of cellular energy metabolism

In 1987, David Carling and Grahame Hardie first demonstrated that apparently two different protein kinases that inhibited enzymes involved in *de novo* fatty acid and cholesterol synthesis (acetyl-CoA carboxylase and hydroxymethylglutaryl-CoA reductase respectively) were in fact the same protein (5). As each enzyme had formerly been shown to be activated by AMP (6, 7), they re-named them both as AMP-activated protein kinase (AMPK) (8). AMPK is now established as the principal energy sensor in eukaryotic cells and is unquestionably one of the most important discoveries in biomedical sciences in the last 30 years. In fact, 3 decades later, AMPK is considered the principal (and probably sole) energy sensor in eukaryotic cells, a concept that has been extended to a more global view in which AMPK has a wide range of effects at the cellular and whole-body levels, regulating, besides metabolism, cell growth, mitosis, apoptosis, cell polarity, autophagy, inflammation, immune function and cancer (4, 9, 10, 11).

## AMPK: the master energy sensor

**Figure 1:**
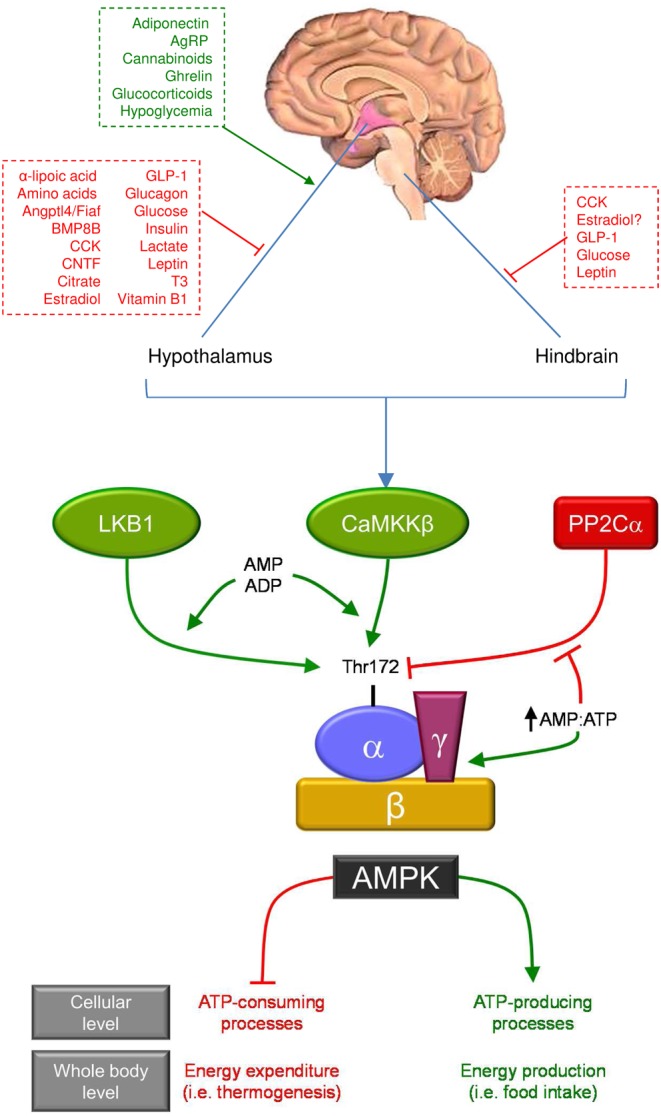
Structure, regulation and the role of AMPK. AMP-activated protein kinase (AMPK) is a heterotrimer complex consisting of a catalytic α subunit and two regulatory subunits, β and γ. AMPK is the downstream component of a kinase cascade that acts as a gauge of cellular energy levels, being activated through increased phosphorylation of Thr172 within the catalytic α subunit by upstream liver kinase B1 (LKB1) and Ca2+/calmodulin-dependent protein kinase kinase β (CaMKKβ). In addition to activation by phosphorylation, AMPK is allosterically activated by AMP, which induces phosphorylation at Thr172 by LKB1 and CaMKKβ and also inhibits dephosphorylation by protein phosphatases such as protein phosphatase 2C alpha (PP2Cα). Other upstream factors modulate AMPK activity (see main text) but have been omitted to simplify the figure. AMPK is a counter-regulatory intracellular switch, switching off (red lines) ATP-consuming processes, while switching on (green arrows) catabolic processes that produce ATP. In several brain areas, such as the hypothalamus, AMPK acts to monitor nutritional and hormonal signals and consequently to regulate energy balance at the whole-body level. Thus, activation of AMPK increases energy (food) intake and decreases energy expenditure (thermogenesis). Red and green lettering represents inhibitory and stimulatory stimuli respectively. AgRP, agouti-related peptide; BMP8B, bone morphogenetic protein 8B; CCK, cholecystokinin; CNTF, ciliary neurotrophic factor; GLP-1, glucagon-like peptide-1; T3, 3,3′,5-triiodothyronine.

AMPK is a highly conserved serine/threonine kinase; certainly, orthologs of AMPK subunits have been found in all eukaryotic kingdoms, including protists, fungi, plants and animals (1, 9). AMPK is a heterotrimer complex comprising a catalytic α (α1, α2) subunit with a conventional serine/threonine protein kinase domain and two regulatory subunits, β (β1, β2) and γ (γ1, γ2, γ3) (Fig. 1), encoded by different genes (1, 4, 9, 12, 13). Briefly, AMPK is activated by phosphorylation of Thr172 of the α subunit, a process that can be allosterically induced by AMP (but not ADP) (14) and catalyzed by several upstream kinases, such as liver kinase B1 (LKB1) (15, 16), the pseudokinase STRAD, the scaffold protein mouse protein-25 (MO25) (17, 18, 19), and calmodulin-dependent kinase kinases (CaMKKs), especially CaMKKβ (20, 21, 22). AMP and ADP not only facilitate phosphorylation at Thr172 by LKB1 and CaMKKβ (14, 15, 16, 23), but also inhibit dephosphorylation by protein phosphatases, such as protein phosphatase 2C alpha (PP2Cα; with AMP being 10-fold more potent than ADP and both being antagonized by ATP) (13, 24, 25). Ca2+-dependent and AMP-dependent pathways occur independently. Thus, a rise in Ca2+ leads to the activation of CaMKKβ, which increases Thr172 phosphorylation and activation of AMPK (26). Finally, a mechanism modulating AMPK independent of AMP and phosphorylation/dephosphorylation processes has been proposed. Cell-death-inducing like-effector A (CIDEA) forms a complex with the β subunit of AMPK, which elicits an ubiquitination-mediated degradation of AMPK, reducing its activity (27) (Fig. 1). A more detailed description of AMPK structure and regulation is beyond the scope of the present review but has been excellently reviewed elsewhere (1, 4, 9, 28, 29, 30).

AMPK is activated in situations that lead to a reduction in intracellular energy levels, such as hypoxia and hypoglycemia, or to those that increase ATP utilization, such as muscle contraction or food deprivation (1, 2, 3, 4, 29, 30). As a result of changes in the ratio of adenine nucleotides, AMPK is phosphorylated leading to ATP-consuming processes, such as fatty acid synthesis being switched off and catabolic processes, such as fatty acid oxidation being switched on. The overall effect of AMPK activation is therefore to produce ATP and restore AMP:ATP and ADP:ATP, allowing balanced rates of catabolism and ATP usage and thus maintaining cellular energy homeostasis (1, 2, 3, 4, 29, 30). Catabolic processes such as mitochondrial biogenesis and autophagy (mitophagy) are turned on (31, 32, 33, 34). AMPK also regulates anabolic processes, with AMPK switching off all anabolic pathways virtually, such as the biosynthesis of lipids, carbohydrates, proteins and ribosomal RNA, when the cellular energy status is diminished (1, 2, 3, 4, 29, 30).

## Hypothalamic AMPK and regulation of food intake

The first evidence implicating hypothalamic AMPK in the modulation of energy balance was demonstrated by David Carling and Caroline Small groups stating that AMPK played a role in the regulation of feeding (35). In their seminal paper, they showed that key hormones controlling food intake, such as leptin and ghrelin, modulated hypothalamic AMPK and that activation of AMPK at this level increased appetite (35). Parallel work by Barbara Kahn and colleagues showed that AMPK is highly expressed in the arcuate (ARC), dorsomedial (DMH), paraventricular (PVH), and ventromedial (VMH) nuclei, as well as in the lateral hypothalamic area (LHA) (36); importantly, they also demonstrated that modulation of hypothalamic AMPK formed part of an adaptive change in the physiological regulation of feeding (36). Thus, fasting increases but refeeding inhibits the AMPK activity in many hypothalamic regions (35, 36, 37). Moreover, at the whole-body level, activation of hypothalamic AMPK leads to increased feeding and weight gain, whereas its inhibition leads to hypophagia and weight loss (36).

In keeping with this physiological evidence, genetic models have demonstrated a key role for hypothalamic AMPK in the modulation of feeding. Initially, it was shown that inhibition of hypothalamic AMPK with AMPK dominant negative (AMPK-DN) isoforms decreases mRNA expression of the orexigenic neuropeptides agouti-related peptide (AgRP) and neuropeptide Y (NPY) in the ARC. However, over-expression of an AMPK constitutively active (AMPK-CA) isoform elevates the fasting-induced expression of AgRP and NPY in the ARC as well as expression of melanin-concentrating hormone (MCH) in the LHA (36). It has been currently reported that AMPK modulates the expression of NPY and pro-opiomelanocortin (POMC) by regulating autophagy (38). These data were taken to suggest that AMPK exerts nucleus-specific effects on feeding control, an idea that was subsequently confirmed by the generation of mice with a conditional deletion of the catalytic subunit of AMPKα2 specifically in POMC or AgRP neurons of the ARC. Interestingly, both mouse models display divergent phenotypes in terms of energy balance; while AMPKα2-POMC KO mice developed obesity due to hyperphagia, AMPKα2-AgRP KO mice developed an age-dependent lean phenotype (39). However, despite the unquestionable role of AMPK in the regulation of feeding, several lines of evidence suggest that the chronic effects of hypothalamic AMPK manipulation on body mass are more closely related to altered energy expenditure than to food intake (see below) (40, 41, 42, 43, 44, 45, 46, 47, 48, 49, 50).

**Figure 2:**
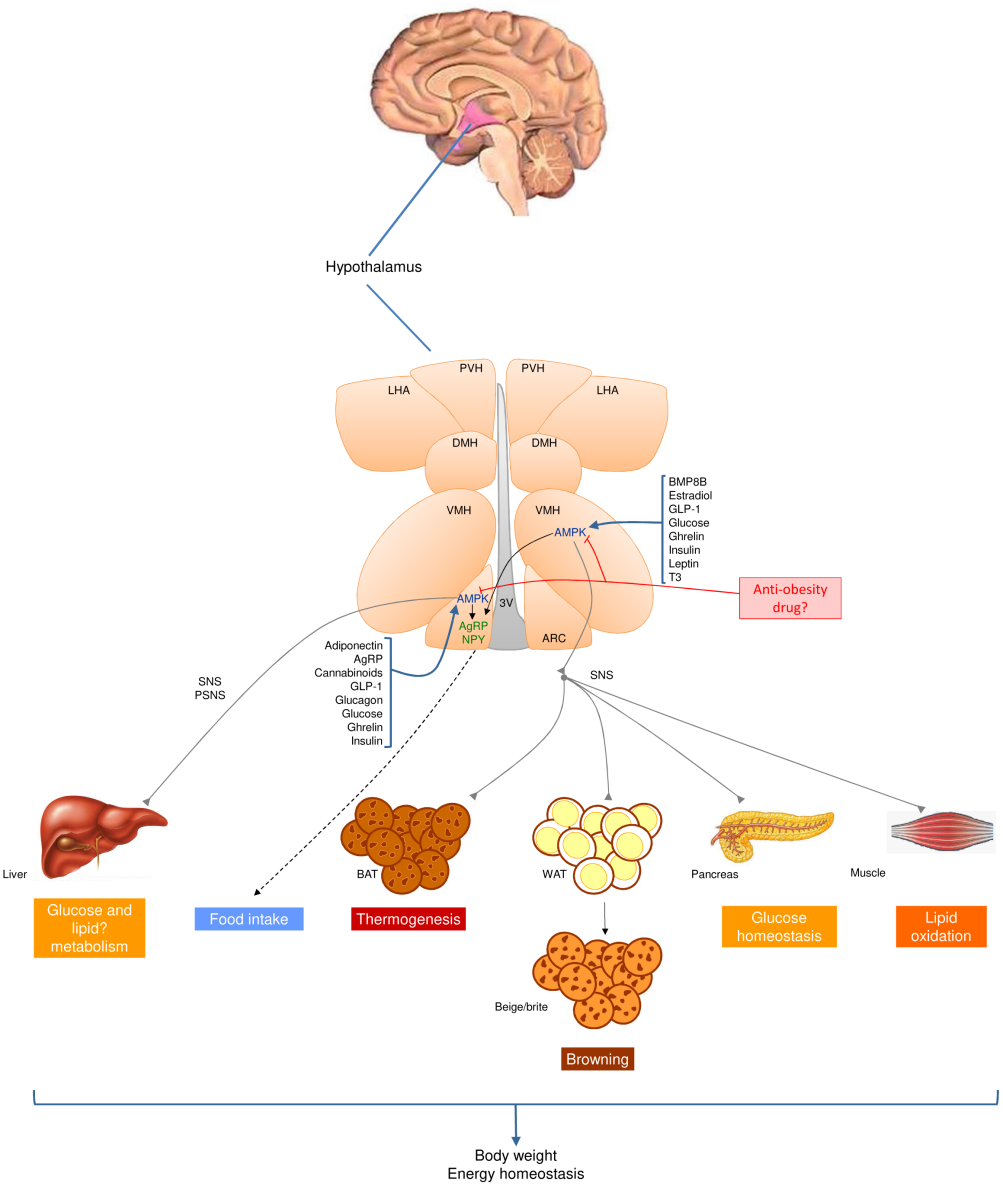
Brain AMPK is a canonical regulator of energy balance. AMP-activated protein kinase (AMPK) acts in the hypothalamus to modulate whole-body energy homeostasis and body weight. AMPK senses several nutritional and hormonal stimuli to regulate food intake, hepatic glucose and possibly lipid metabolism, brown adipose tissue (BAT) thermogenesis, browning of white adipose tissue (WAT), glucose homeostasis and lipid and glycogen synthesis in skeletal muscle. The actions of hypothalamic AMPK on peripheral tissues/organs are mediated by specific regulation of the sympathetic (SNS) and parasympathetic nervous systems (PSNS). The fact that inhibition of hypothalamic AMPK leads to anorexia and increased thermogenesis (and therefore elevated energy expenditure) makes it an interesting target for drug development, with its potential for controlling both sides of the energy balance equation. 3V, third ventricle; AgRP, agouti-related peptide; ARC, arcuate nucleus of the hypothalamus; BMP8B, bone morphogenetic protein 8B; DMH, dorsomedial nucleus of the hypothalamus; GLP-1, glucagon-like peptide-1; LHA, lateral hypothalamic area; NPY, neuropeptide Y; PVH, paraventricular nucleus of the hypothalamus; T3, 3,3′,5-triiodothyronine; VMH, ventromedial nucleus of the hypothalamus.

Notably, most actions of AMPK in the hypothalamus relate to mediation of hormonal effects. Both orexigenic and anorexigenic hormones converge on hypothalamic AMPK to modulate appetite. The consensus view is that while the main anorexigenic factors inhibit hypothalamic AMPK, the vast majority of orexigenic hormones activate it (40, 41, 42, 43, 44, 45, 46, 47, 48) (Figs 1 and 2). For example, physiological appetite inhibitors such as leptin (35, 36, 51), insulin (36, 52), glucagon-like peptide-1 (GLP-1) (46, 53), estradiol (E2) (45, 54) and ciliary neurotrophic factor (CNTF) (55), inhibit hypothalamic AMPK. In contrast, activation of hypothalamic AMPK is caused by orexigenic signals such as adiponectin (56, 57), glucocorticoids (58), ghrelin (35, 37, 59, 60, 61, 62), cannabinoids (59, 63) and AgRP (36). Resistin (RSTN), despite its anorectic effect, activates hypothalamic AMPK (64).

The fact that hypothalamic AMPK has emerged as a key modulator of food intake is of interest because several pharmacological factors with well-established impacts on feeding, such as melanocortin receptor agonists (including melanotan II; MTII) and nicotine, exert their actions by inhibiting hypothalamic AMPK (36, 44, 65). In contrast, antipsychotic drugs (APDs), such as olanzapine, well known for their orexigenic and obesity-prone properties, activate hypothalamic AMPK (66, 67, 68, 69). Overall, the evidence suggest that central AMPK is a potential target for the treatment of obesity, an idea that is reinforced by AMPK’s effects on energy expenditure (40, 41, 42, 43, 44, 45, 46, 47, 48, 49, 50) (see below).

## Hypothalamic AMPK and regulation of thermogenesis

The hypothalamus also plays a major role in the regulation of brown adipose tissue (BAT) thermogenesis through the sympathetic nervous system (SNS). BAT is activated by increased firing of sympathetic neurons, leading to release of noradrenaline and activation on β3-adrenergic receptors (β3-AR) (70, 71, 72, 73, 74). Within the hypothalamus, the VMH was the first hypothalamic location to be identified as important in BAT thermogenic activity (75). The VMH is connected to other brainstem regions linked to the regulation of BAT, such as raphe pallidus (RPa) and the inferior olive (IO), which control sympathetic activation of BAT (70, 71, 72, 73).

Recent evidence has demonstrated that hypothalamic AMPK is a major regulator of BAT thermogenesis through its modulation of the SNS. By analyzing the central effects of thyroid hormones (THs) on energy homeostasis, we demonstrated that central specific administration of 3,3′,5-triiodothyronine (T3) within the VMH (but not in the ARC) promotes a profound thermogenic response that is associated with decreased AMPK activity in the VMH and, importantly, elevated sympathetic firing in brown fat (41, 49, 76, 77). Notably, targeted administration of adenoviruses harboring AMPK-CA isoforms to the VMH reduced the activation of BAT and prevented the weight loss, which is usually associated with central T3 action, in a feeding-independent but uncoupling protein 1-dependent manner (41, 49). The significant aspect of such an integrative mechanism is that it constitutes a canonical circuit that is non-exclusive for THs and mediates the effects of other thermogenic molecules. For example, central administration of E2 inhibits AMPK through estrogen receptor alpha (ERα), selectively in the VMH (but not in the ARC), leading to feeding-independent activation of thermogenesis in brown fat through the SNS (45, 78). Again, virogenetic activation of AMPK in the VMH (but not in the ARC) prevented an E2-induced increase in BAT-mediated thermogenesis and weight loss (45). Notably, fluctuations in E2 levels during the estrous cycle and pregnancy also modulate this integrated AMPK network, indicating its physiological relevance (45, 78). Current evidence shows that bone morphogenetic protein 8B (BMP8B) acts centrally and that its thermogenic effect is dependent on the activation status of AMPK in the VMH. In fact, BMP8b-induced thermogenesis can be completely prevented by AMPK-CA isoforms within the VMH (43, 50), as well as with pharmacologic antagonist or genetic deletion of orexin (OX) in the LHA (50). If OX neurons in the LHA also mediate THs and/or E2 is currently unknown, but seems likely when considering the similitude in thermogenic response and the known neuroanatomical connections.

Together, these findings demonstrate that hormonal regulation of the VMH AMPK-(LHA OX)-SNS-BAT axis is an important determinant of energy balance (47, 48) and suggest that dysregulation of this axis might account for common changes in energy homeostasis associated with alterations in thyroid and ovarian status, together with impaired BMP8B function (41, 43, 45, 78). In this context, recent data have also indicated that VMH AMPK could be an interesting target for the treatment of obesity. For example, nicotine, the main bioactive compound of tobacco, stimulates thermogenesis and weight loss through AMPK in the VMH (44, 65). More importantly, liraglutide, a GLP-1 agonist currently used clinically for the treatment of type 2 diabetes (T2D), exerts a potent central thermogenic action, in addition to inducing browning of WAT, by modulating AMPK specifically in the VMH among tested hypothalamic sites (46). Again, that effect is accompanied by significant weight loss (46). Further studies are necessary to address the sub-cellular mechanisms and neuronal networks involved in the VMH AMPK-(LHA OX)-SNS-BAT axis.

## AMPK modulators and metabolic disorders

AMPK has become a potential therapeutic target in metabolic diseases involving impaired eating behaviors, including obesity, T2D and some lipodystrophies. Although activation of AMPK can be expected to lead to a reduction of ectopic lipid storage in liver and muscle with an improvement in insulin sensitivity, it can also affect energy homeostasis in a tissue-specific manner. AICAR (5-aminoimidazole-4-carboxamide ribonucleotide) was one of the first-described direct activators of AMPK (79). However, despite its improving glucose tolerance and reducing circulating triglycerides (TG) and free fatty acids (FFA) (80), its poor bioavailability and short half-life make it unlikely to be used in humans (81). Other direct AMPK activators, such as 991 and A-769662, are more potent and some studies have found reductions in plasma glucose and lipid levels (82). However, side effects related to cell cycle progression (83) also make these direct AMPK activators unlikely to be used therapeutically.

Reduced ectopic lipid storage and increased insulin sensitivity can be driven by increasing glucose uptake by skeletal muscle (33, 84) or inhibiting glucose production in the liver (85). Various AMPK activators are currently used to ameliorate high glucose levels in T2D. Metformin, a synthetic biguanide, activates AMPK indirectly by inhibiting the mitochondrial respiratory chain (86, 87). Metformin reduces glycated hemoglobin HbA1c by 2% in T2D patients, with very few side effects, simultaneously reducing the risk of cardiovascular diseases (88) and certain types of cancer (89). Its main action is to inhibit the hepatic glucose production (90), which appears to be LKB1 dependent and therefore is mediated by an indirect activation of AMPK, as LKB1-null animals do not have reduced levels of glucose (18, 91). Recent reports have also shown that metformin exerts its AMPK-independent effects in the liver (92). In contrast, mice with acetyl-CoA carboxylase (ACC) mutations are refractory to the lipid and glucose-reducing effects of metformin (93). In addition to metformin, thiazolidinedione compounds, such as rosiglitazone and pioglitazone, also produce a dramatic increase in AMP in skeletal muscle, which results in a rapid activation of AMPK (94). These drugs also seem to activate AMPK indirectly, through peroxisome proliferator-activated receptor-gamma (PPARγ), which in turn stimulates adiponectin secretion (95). Other drugs, such as liraglutide and exenatide (the synthetic form of exendin-4), have been designed to mimic the action of GLP-1 to increase insulin sensitivity (96). Liraglutide has been reported to have opposing effects on AMPK. Although it increases AMPK phosphorylation in endothelium (reducing inflammation) (97, 98), heart (99), liver and muscle (enhancing insulin sensitivity) (100, 101), and WAT (102), it decreases AMPK phosphorylation in pancreatic β cells (leading to their proliferation) (103) and in the hypothalamus (leading to anorexia and increased BAT thermogenesis) (46). Exendin increases hepatic AMPK phosphorylation, thus ameliorating steatosis (104).

As well as these Beside synthetic compounds, there are naturally occurring molecules that have also been shown to elicit metabolic benefits through AMPK activation. Resveratrol, for example, found in the skin of red grapes, has been shown to activate AMPK indirectly and increase muscle glucose uptake (105), possibly by increasing intracellular Ca2+ levels and thus activating CaMKKβ (106). Resveratrol has also been reported to reduce lipid accumulation in the liver in an AMPK-dependent manner, as these effects are blunted when AMPK is genetically blocked (107). Quercetin, the most abundant flavonoid, is thought to have metabolically protective roles. Thus, it has been reported to exert an anti-adipogenic action mediated by activation of AMPK and its substrate ACC (108). In the same context, there is evidence to suggest that quercetin positively affects glucose metabolism in both liver and muscle through an insulin-independent mechanism involving AMPK activation (109). Quercetin also appears to have beneficial effects by protecting against cholesterol-induced neurotoxicity (110, 111). Other plant-derived compounds, such as rooibos and berberine, improve glucose homeostasis and reduce cholesterol levels, with these benefits being attributed to activation of AMPK in liver (112, 113), muscle (114) and adipose tissue (113, 115).

It is notable that patients with metabolic disorders, such as T2D, insulin resistance and obesity are at an increased risk of developing cancer (116). Since AMPK activation inhibits anabolism leading to cell arrest, it is logical to speculate that AMPK might prevent tumor progression. As AMPK mutations leading to tumorigenic processes are rare, in humans, it seems more likely that defective upstream effectors or downstream targets of AMPK will be found to be causative. In this context, inactivation of LKB1 induces activation of mTORC1, which promotes cell growth and proliferation (18, 91), whereas mutations in LKB1 prevent activation of AMPK, causing Peutz–Jeghers syndrome, which is a risk factor for developing cancer (18). It has been shown that inactivation of AMPK enhances the aerobic glycolysis that is likely to cause activation of oncogenes and inhibition of tumor suppressors (117). Thus, activation of AMPK has been suggested as a possible therapy in cancer. Indeed, evidence suggests that the AMPK activator metformin can reduce the tumor size (89). In contrast, high levels of pACC have been found in prostate cancer cells, implicating activated AMPK in prostate cancer (118). Since AMPK activation is inhibited when energy levels are in a normal fed state, continued AMPK activation might be essential for the survival of cancer cells. Further studies are necessary to understand the role of AMPK in the cell cycle and in the development of cancer. In this context, very recent evidence shows that AMPK plays a major role in regulating glycolysis and cell survival in response to mitophagy during mitotic arrest (11).

## Is hypothalamic AMPK a realistic therapeutic target against obesity?

Obesity causes thousands of deaths per year worldwide, directly and indirectly due to comorbidities including cancer, cardiovascular disease and T2D, and yet, it is the most preventable epidemic (96, 119, 120, 121). However, despite significant investments in education and public engagement, government-led policies are relatively ineffective. This is shown in the World Health Organization (WHO)’s latest report, which states that globally, 13% of adults are obese. In healthy individuals, maintaining normal weight is a matter of lifestyle. However, such apparent simplicity also necessitates an understanding of how the body manages what, how, when and why we eat, as well as how we expend calories. Each of these functions is carried out by different hormones and peptides that respond to the various physiological states occurring in arousal and sleep, with some having circadian rhythms.

Data accrued over the last decade have demonstrated an unequivocally key role of hypothalamic AMPK in the regulation of both parts of the energy balance equation, i.e. feeding and energy expenditure (40, 42, 47, 48). Activation of AMPK in peripheral organs is one of the mechanisms of the widely used antidiabetic drug, metformin (for an extensive review see (87). However, central activation of AMPK would not give the best outcome in treatment for obesity as it would increase feeding while decrease BAT thermogenesis due to its differential regulation in the periphery (122) and centrally (36, 37, 40, 42, 47, 48). Inhibition of AMPK in peripheral tissues would also have deleterious consequences, worsening insulin resistance and developing diabetes. However, the best strategy would be to specifically target hypothalamic AMPK that also looks to be a highly complex task. The use of nanoparticle or exosome approaches (123) might be an option, but directing them to specific hypothalamic populations, e.g. to AMPK neurons in the VMH (whose inhibition could promote anorexia, and increase thermogenesis and weight loss) (Fig. 2), seems challenging. Another alternative might be optogenetic modulation of hypothalamic AMPK neurons, which has already been elegantly achieved in rodents (124). However, the implementation of optogenetics for hypothalamic intervention in humans also seems a distant possibility. Perhaps a more realistic strategy would be to use peptide conjugates (with other peptides or steroid hormones) (120, 121, 125, 126, 127) in a targeted approach. For example, a chimera containing GLP-1 plus an estrogen, (125) or glucagon plus T3 (127), would allow quite a precise targeting of AMPK neurons in the VMH, although the fact that other neuronal populations would be affected (128) would limit specificity.

Overall, such restraints raise some doubts about the translatability of the data into clinical practice. Even if hypothalamic AMPK can be specifically targeted, other questions and potential problems emerge. In my view, the most relevant issue to address is that of the long-term consequences of targeting AMPK in the brain. Considering the central role of AMPK on lipid and glucose metabolism, how would neurons respond to sustained AMPK inhibition? Would they survive? In this sense, recent data have shown that impaired lipid metabolism in neurons leads to lipotoxicity, endoplasmic reticulum stress, and leptin and insulin resistance (129, 130, 131, 132, 133, 134, 135), which would be a deleterious side effect. Would their modulation affect other hypothalamic-mediated physiological processes such as regulation of endocrine axes? (61). A substantial amount of work will be necessary to address these questions and to understand the molecular and neural mechanisms upstream and downstream of central AMPK fully, itself a fascinating endeavor for the years to come.

## Declaration of interest

The author declares that there is no conflict of interest that could be perceived as prejudicing the impartiality of the research reported.

## Funding

The research leading to this work has received funding from the European Community’s Seventh Framework Programme (FP7/2007–2013) under grant agreement n° 281854-the *ObERStress* project, Xunta de Galicia (2015-CP079 and 2016-PG068), MINECO co-funded by FEDER (SAF2015-71026-R and BFU2015-70454-REDT/*Adipoplast*). CIBER de Fisiopatología de la Obesidad y Nutrición is an initiative of ISCIII.
